# Circulating Brain-Derived Neurotrophic Factor, Antioxidant Enzymes Activities, and Mitochondrial DNA in Bipolar Disorder: An Exploratory Report

**DOI:** 10.3389/fpsyt.2020.514658

**Published:** 2020-09-11

**Authors:** Dong Wang, Hong Li, Xiangdong Du, Jun Zhou, Liu Yuan, Honghong Ren, Xiaonan Yang, Guangya Zhang, Xiaogang Chen

**Affiliations:** ^1^ Department of Geriatric Psychiatry, Suzhou Mental Health Center, Suzhou Guangji Hospital, the Affiliated Guangji Hospital of Soochow University, Suzhou, China; ^2^ Department of Psychiatry, the First Affiliated Hospital of Zhengzhou University, Zhengzhou, China; ^3^ Department of Psychiatry, the Second Xiangya Hospital of Central South University, China National Clinical Research Center on Mental Health Disorders, China National Technology Institute on Mental Disorders, Hunan Technology Institute of Psychiatry, Hunan Key Laboratory of Psychiatry and Mental Health, Mental Health Institute of Central South University, Changsha, China

**Keywords:** bipolar disorder, brain-derived neurotrophic factor, mitochondrial DNA, oxidative stress, manganese superoxide dismutase

## Abstract

**Aim:**

Accumulated evidence indicates that neurotrophin deregulations, oxidative stress injury, and mitochondrial dysfunction have been involved in bipolar disorder (BD); however, their real roles in BD are unclear. Investing the possible interaction between three systems is worthwhile understanding this complex process.

**Methods:**

We measured plasma brain-derived neurotrophic factor (BDNF) level, leukocytes mitochondrial DNA copy number (mtDNAcn), and activities of antioxidant enzymes in BD patients (n = 97) and healthy controls (n = 31). Analysis of variance and linear regression analyses were performed to explore the interaction between mtDNAcn, antioxidant enzymes, and BDNF.

**Results:**

Compared with healthy controls, there were significant decreases of glutathione peroxidase activity, BDNF levels, and mtDNA content, significant increases of manganese superoxide dismutase (MnSOD) activity among BD patients (all p < 0.05). Regression analysis showed MnSOD activity had a moderate effect on BDNF (beta = 0.23, t = 8.5, p = 0.001). Copper zinc SOD and total SOD activity were significantly correlated with Hamilton Depression Scale scores in depressive patients (r = −0.38, p = 0.013; r = −0.35, p = 0.022). Unexpectedly, we observed no significant correlation between mtDNA content and BDNF in BD patients (p > 0.05).

**Conclusion:**

The findings coincide with our hypothesis that abnormal antioxidant enzymes, mtDNAcn, and peripheral BDNF may be involved in the course of BD. There were significant correlations between peripheral BDNF, antioxidant enzyme activities and mtDNAcn, suggesting that oxidative stress, mitochondrial function, and BDNF may influence each other in BD.

## Introduction

Bipolar disorder (BD) is a debilitating and progressive psychiatric disorder, characterized by recurrent episodes of mania and depression, affecting more than 1% of the population worldwide (1). BD is considered one of the leading causes of disability among adults (2), and causes a heavy burden on patients, their families, and society (3). Despite the clinical importance of BD, the neurobiological underpinning of BD remains unclear. It is known that BD has been supposed to be not only a psychiatric disease, but rather a multi-system disorder (4), involving multiple factors, such as genetic, environmental, and social background; neurotrophin deregulations, oxidative stress injury, and mitochondrial dysfunction (5, 6). These potential biomarkers are very important to understand the pathophysiology of BD, and develop new treatment strategies.

Brain-derived neurotropic factor (BDNF), an important member of neurotrophins, plays a crucial role in neurodevelopment, neuronal plasticity, and survival. Meta-analysis has shown that peripheral BDNF levels are significantly decreased in manic and depressive states of BD (6). Furthermore, BDNF levels could be restored after psychiatric treatment (7), indicating that BDNF may be a promising biomarker for disease activity in BD (6, 8, 9). Intact BDNF as a small-secreted protein, could cross the blood-brain barrier (10), so BDNF levels in the blood may reflect similar levels of BDNF in the brain (11). Although BDNF has been reported to be involved in the pathology of BD, the role of BDNF in the genesis and development of BD is still not well understood.

Mitochondria are key cellular organelles that are responsible for generating energy through oxidative phosphorylation, and related to cellular growth, resilience, death, neuroplasticity, and neuronal activity (12, 13). Mitochondrial dysfunction and oxidative stress have been shown to be involved in BD (14). Mitochondrial DNA (mtDNA) is vulnerable to oxidative or genotoxic injury because of a lack of protection by histones, causing large DNA mutations and functional impairments. Genetic, molecular, and postmortem studies have confirmed that the impairments of neural systems in BD can be caused by mitochondrial deregulations (14). Oxidative stress involves an oxidant-antioxidant imbalance, which may also play important roles in BD pathophysiology. Convergent evidence indicates systemic changes of oxidative stress agents as well as antioxidant agents during the development of BD (15). For example, superoxide dismutase (SOD) and glutathione peroxidase (GPx), two key enzymes of the antioxidant system, were reported to be related to mood episodes. Although several clinical studies found abnormal levels of SOD and GPx in manic and depressive patients, when compared with healthy controls, inconsistent or even contradictory data has been frequently reported (15–17).

Growing evidence from animal and *in vitro* studies has suggested that oxidative stress, mitochondrial function, and BDNF have a complex and reciprocal relationship. Mitochondrial complex I is responsible for oxidative phosphorylation and generating ATP in mitochondria, and is related to the levels of intra and extracellular BDNF (18, 19). BDNF has also been reported to improve mitochondrial respiratory coupling by providing neuroprotection after its interaction with ATPase (19, 20). Several studies have shown increased oxidative stress and decreased BDNF levels, indicating that BDNF may play a protective role against oxidative damage in neurons (21, 22). He et al. showed that BDNF increased the antioxidant capacity of cell by selectively enhancing the expression levels of MnSOD (22). Although abnormal BDNF levels, enzymes activities, and mtDNA content (a biomarker, which reflects the mitochondrial function) were found in BD, there have been few studies on the relationship between BDNF, oxidative stress, and mtDNA in patients with BD. Only one study has reported that serum BDNF levels are related with the levels of antioxidant enzymes including SOD, and GPx in BD (23). Two studies reported that, patients with BD exhibited a negative association between serum BDNF levels and lipid peroxidation (24, 25). To our best knowledge, no study has been performed to examine the relationship between mtDNA content and BDNF in individuals with BD. Despite the inconsistencies, the above findings indicated that oxidative stress damage and mitochondrial dysfunction may be linked to BDNF levels to disrupt cellular resilience in BD.

Clarifying the relationship of mitochondrial biology, oxidative stress, and BDNF in mood disorders will provide us more clues to the mechanism of BD. In this study, we measured the leukocyte mtDNA content, plasma antioxidant enzymes including manganese SOD (MnSOD), copper zinc SOD (CuZnSOD), total SOD, and GPx, and BDNF levels to explore i) whether these biomarkers were altered in acute patients with BD; ii) whether there was a correlation among BDNF levels, SOD enzymes activities, mtDNA content, and clinical symptoms. We hypothesized that abnormal antioxidant enzymes activities, mtDNAcn, and peripheral BDNF levels occurred simultaneously in BD patients, and changes of antioxidant enzymes and mtDNA content were associated with the levels of BDNF.

## Materials and Methods

### Subjects and Assessments

Patients with BD I type, including 51 manic and 46 depressive state individuals were recruited from the Suzhou Guangji Hospital (Jiangsu Province, China) during the period from July 2017 to May 2018. Inclusion criteria for BD patients were adults aged 18–65 years and defined by the occurrence of no less than two mood episodes of BD. All BD-I patients were diagnosed by more than two psychiatrists with expertise according to the Structured Clinical Interview for DSM-IV Axis I Disorders (SCID-IV). Types and doses of medication were recorded, but information on the duration of treatment was lacking. The affective symptoms were assessed using the Young Mania Rating Scale (YMRS), Hamilton Depression Scale (HAMD), and Clinical Global Impression-Bipolar Disorder-Severity of Illness Scale (CGI-BP-S). Thirty-one healthy participants were enrolled by advertisement. The volunteers were screened for psychiatric disorders using SCID-IV, non-patients edition (SCID-NP). Inclusion criteria for healthy control subjects were adults aged 18–65 years, with no history of mental disorders in the volunteers and their first-degree relatives. Exclusion criteria of all subjects were as follows: other mental illness, neurological diseases, substance abuse, several medical conditions including diabetes, common metabolic diseases, and cardiovascular diseases. All participants and/or their guardians read and signed the informed consent form. The study was approved by the ethics committee of Suzhou Guangji Hospital.

### Biomarker Assay

Six milliliters of blood was withdrawn into ethylenediamine tetraacetic acid tubes from each fasted participant before taking medicine, between 7:00 and 9:00 am. For plasma isolation, 3 ml of blood was centrifuged at 3,500 *g* for 10 min. Three milliliters of venous blood and isolated plasma were kept at −80°C for further analyses. BDNF levels were evaluated with a duplicate enzyme immunoassay according to the manufacturer’s instructions (Mlbio, Shanghai, China). The minimal limit of detection of the assay was 0.1 ng/ml. The inter-assay coefficient of variation (CV) was < 7% and the intra-assay CV was < 6%. Plasma MnSOD, CuZnSOD, total SOD (TSOD), and GPx activities were measured in triplicate by the spectrophotometric method (26, 27) using commercially available assay kits (Mlbio, Shanghai, China). The lower limit of detection of SOD (MnSOD, CuZnSOD, TSOD) and GPx activities were 5.0 and 10.0 U/ml, respectively. The inter-assay and intra-assay CV ranged from 2.7 to 6.1%. All measurements were performed by the same technician. (See details in the **Supplementary Methods**).

### Mitochondrial DNA Content Measurement

The detailed measurement of mtDNA copy number (mtDNAcn) was described in our previous research (28, 29). In brief, genomic DNA was extracted following the protocol using the QIAmp DNA Mini Kit (Qiagen, Hilden, Germany). The relative mtDNAcn was determined by the fluorescence-based quantitative polymerase chain reaction (qPCR). The primers for the mitochondrial gene (NADH dehydrogenase 1 gene, *ND1*) were as follows: F (5’-CCCTAAAACCCGCCACATCT-3’) and R (5’-GAGCGATGGTGAGAGCTA-AGGT-3’). The primers for the reference nuclear gene (human hemoglobin beta gene, *HBB*) were F (5’-GTGCACCTGACTCCTGAGGAGA-3’) and R (5’-CCTTGATACCAACCTGCCCAG-3’). All experiments were conducted in triplicates. The amplification of qPCR was performed using the standard curve assay. Efficiencies of the amplifications were between 93.4 and 108.2%. The intra-assay coefficient of variation (CV) was < 3.7%, and the inter-assay CV was < 3.3%. (See details in the **Supplementary Methods**).

### Statistical Analysis

The Kolmogorov-Smirnov test was used to test the normality of continuous variables. The variables with normality are shown as the mean ± SD; whereas the skewed distributed variables are presented as the median and interquartile ranges. The data with non-normality were log-transformed into a normal distribution. Differences of values among multiple groups were analyzed using chi-square tests for categorical variables, Student’s *t* tests, and one-way analysis of variance for continuous variables. Bonferroni corrections were performed to correct for multiple comparisons. Pearson’s correlations and partial correlations were conducted to examine the relationship between variables. A multiple stepwise linear regression analysis was performed with the BDNF as the dependent variable. Variables with a significant association with BDNF were imputed as independent. Based on a recent meta-analysis of peripheral BDNF in BD, the duration of illness, symptom severity (CGI-BP-S scores), and age were added as adjustment variables (3). All the data were analyzed using SPSS statistical software for Windows, version 22.0 (SPSS, Chicago, IL, USA). Two-side tests and adjusted p < 0.05 were considered significant.

## Results

**Table 1** shows the demographic, clinical, and pharmacologic data of individuals. All participants were young, with mean ages of 26.90 ± 7.26 (manic group), 27.07 ± 9.12 (depressive group), and 24.51 ± 4.10 (healthy controls). No significant differences in the three groups were found in terms of age, gender, smoking, and body mass index (BMI). The education grade of the healthy group was slightly higher than patients groups; however it was not statistically significant (p = 0.081). The manic group displayed serious manic symptoms, whereas the depressed group showed noticeable depressive symptoms. Other clinical characteristics were similar between the two groups (manic and depressive groups).

**Table 1:** Clinical and demographic characteristics of the study population.

	Bipolar patients		Health control	Statistic
	Manic n = 51	Depression n = 46	n = 31	*p*
Age, mean (SD)	26.90 (7.26)	27.07 (9.12)	24.51 (4.10)	0.28
Sex male	22	26	16	0.41
Female	29	20	15	
Years of education, mean (SD)	12.73 (2.88)	12.36 (3.19)	13.84 (2.78)	0.081
BMI, mean (SD)	21.38 (2.44)	21.81 (1.83)	20.67 (2.87)	0.12
Smoking, n (%)	18 (35.29)	17 (36.96)	11 (35.48)	0.98
Medicine				
Antipsychotics, n (%)	47 (90.16)	37 (80.43)		0.13
Anticonvulsants, n (%)	31 (60.78)	15 (32.61)		0.41
Benzodiazepines, n (%)	18 (35.92)	15 (32.61)		0.83
Lithium, n (%)	32 (62.75)	25 (54.35)		0.42
CPZ equivalents median (interquartile range)	235.06 (200, 266)	226.5 (200, 266)		0.63
Clinical characteristics				
Duration of illness, years, mean (SD)	5.94 (5.02)	5.25 (5.04)		0.50
YMRS, mean (SD)	34.47 (6.36)	5.36 (4.50)		<0.001
HAMD, mean (SD)	4.47 (2.31)	27.80 (6.65)		<0.001
CGI-BP-S, mean (SD)	5.41 (1.11)	5.22 (1.23)		0.42
Number of previous episodes, mean (SD)	3.51 (1.71)	3.57 (1.70)		0.87

SD, standard deviation; BMI, body mass index; YMRS, Young Mania Rating Scale; HAMD, Hamilton Depression Rating Scale; CGI-BD-S, Clinical Global Impression-Bipolar Disorder-Severity of Illness Scale; CPZ, chlorpromazine.

**Figure 1:**
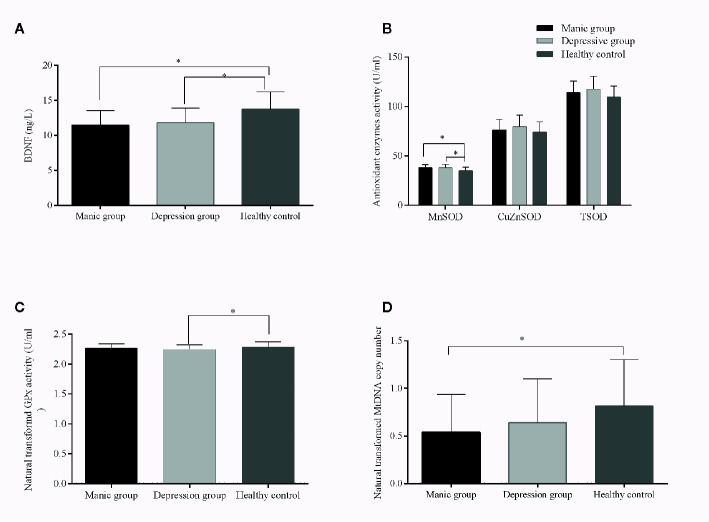
Brain-derived neurotrophic factor (BDNF) levels and TSOD, MnSOD, CuZnSOD, GPx enzyme activities, and mitochondrial DNA (mtDNA) copy number from patients with bipolar disorder and from healthy controls. The results are expressed as the mean+SD, * *p* ≤ 0.05 (controlled for age, gender, education, and BMI). Compared with controls, **(A)**: lower levels of BDNF in manic and depressive patients; **(B)**: increased MnSOD activities in manic and depressive patients; **(C)**: decreased GPx activity in depressive patients; **(D)**: lower mtDNAcn in manic patients.

Analysis of variance showed that the patient groups presented higher MnSOD activity than healthy volunteers (p < 0.05). After adjustments for age, gender, education, and BMI, the differences were still significant (p = 0.004) (**Table 2**). *Post hoc* test showed that there were significantly increased MnSOD levels in manic and depressive patients compared with controls (manic group *vs.* controls, adjusted p < 0.001, depressive group *vs.* controls, adjusted p = 0.01, **Figure 1B**), whereas there was no significant difference between the manic and depressed groups (p > 0.05, **Figure 1B**). The patient groups did not differ in their CuZnSOD activities when compared with controls (p = 0.26). Differences of TSOD (MnSOD plus CuZnSOD) activity among the three groups also did not reach statistical significance (p = 0.091), after adjusting for covariates. GPx activity was reduced in BD patients. When controlling for covariates, the differences of GPx levels were still significant (p = 0.008). *Post hoc* test showed that only depressive group had a decreased GPx activity (p < 0.05, **Figure 1C**). The activity of GPx did not alter when comparing manic group and healthy controls (p > 0.05, **Figure 1C**).

**Table 2:** Antioxidant enzymes activities, brain-derived neurotropic factor (BDNF) levels, and mitochondrial DNA copy number (mtDNAcn) in bipolar disorder (BD) patients and healthy controls.

Markers	Bipolar disorder		Healthy controls	Statistic	*p*	Adjust p*
	Manic (n = 51)	Depression (n = 46)	N = 31	F		
MnSOD (U/ml)	38.10 (2.93)	37.96 (3.52)	35.07 (3.59)	9.38	<0.001	0.004
CuZnSOD (U/ml)	75.96 (69.33, 86.15)	81.32 (70.86, 89.34)	73.57 (64.67, 82.66)	2.54	0.083	0.26
GPx (U/ml)	189.96 (27.35)	179.18 (29.95)	197.31 (34.76)	3.54	0.032	0.008
TSOD (U/ml)	133.93 (11.76)	117.50 (13.23)	109.56 (11.29)	3.93	0.022	0.091
BDNF (ng/ml)	11.47 (2.07)	11.80 (2.09)	13.70 (2.50)	10.86	<0.001	0.001
MtDNAcn	1.64 (1.39, 2.07)	1.96 (1.28, 2.32)	2.44 (1.75, 3.21)	3.66	0.028	0.045

MnSOD, manganese superoxide dismutase; CuZnSOD, copper zinc superoxide dismutase; TSOD, total SOD; GPx, glutathione peroxidase; BDNF, brain-derived neurotropic factor; mtDNAcn, mitochondrial DNA copy number; BD, bipolar disorder. *Adjusting for age, gender, education, and body mass index,*p ≤0.05 were considered to be statistical significant from one-way analysis of variance for all three groups; numbers in parentheses referred to standard deviation or Interquartile range of parameters values.

The two patient groups had lower levels of BDNF than health controls (manic *vs.* controls, p < 0.001; depressive *vs.* controls, p = 0.005, **Figure 1A**). Besides, no significant differences in BDNF levels were observed in manic and depressive groups (p > 0.05, **Figure 1A**). MtDNA content of BD patients was significantly decreased compared with healthy controls (p = 0.028). When the covariates were included, significant differences remained (p = 0.045). Paired comparison indicated, only the mtDNAcn of manic group had a significant decrease when compared with healthy controls (adjusted p = 0.03, **Figure 1D**), whereas negative results were found for other comparisons (manic *vs.* depressive, adjusted p = 0.56; depressive *vs.* controls, adjusted p = 0.27, **Figure 1D**).

**Figure 2:**
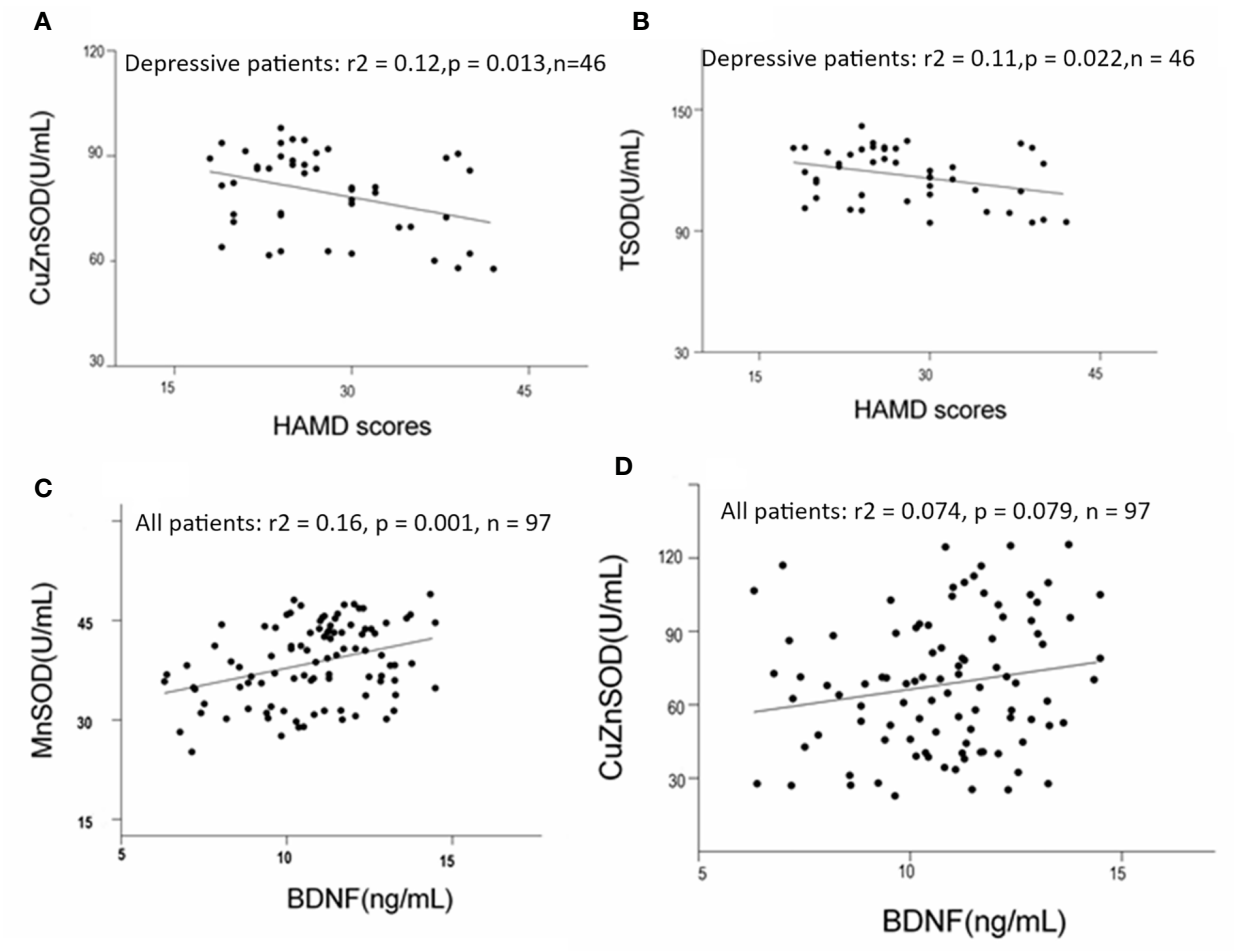
**(A, B)** Correlations among HAMD scores and antioxidant enzymes activities in bipolar disorder patients (adjusted for age, sex, BMI, and smoking, using partial correlation test). **(C, D)** Correlation between BDNF levels and antioxidant enzymes in bipolar disorder (adjusted for duration of illness, symptom severity, and age, using linear regression analyses). HAMD, Hamilton Depression Scale; BMI, body mass index.

For the depressed group, CuZnSOD and TSOD activities were significantly correlated with HAMD scores (r = −0.35, p = 0.017; r = −0.329, p = 0.026). To further identify the relationship between these antioxidant enzyme activities and clinical characteristics, partial correlation analyses were performed (adjusted for age, sex, BMI, and smoking). The results also indicated that there were negative correlations between CuZnSOD activity, TSOD activity, and HAMD scores (r = −0.38, p = 0.013; r = −0.35, p = 0.022, respectively) (**Figures 2A, B**). There were no significant associations between HAMD scores and MnSOD activity (r = −0.078, p = 0.61), GPx activity (r = −0.03, p = 0.98), MtDNAcn (r = −0.23, p = 0.13), or BDNF (r = 0.04, p = 0.98). Regarding the manic group, we also did not find any significant correlations between YMRS scores and CuZnSOD activity (r = −0.061, p = 0.67), MnSOD activity (r = 0.14, p = 0.34), GPx activity (r = 0.013, p = 0.93) as well as BDNF (r = 0.012, p = 0.93). (All correlations for the biomarkers and clinical symptom scores were listed as **Supplementary Results**).

No significant correlations were found between mtDNA content and BDNF (r = −0.062, p = 0.54), or between GPx activity and BDNF (r = −0.063, p = 0.53) in all acute patients. Circulating BDNF was positively associated with MnSOD and CuZnSOD activities (r = 0.41, p < 0.001, r = 0.24, p = 0.005), (**Figures 2C, D**). Linear regression analyses were conducted to identify influencing factors for BDNF levels. The results showed MnSOD remained significant with a moderate effect on BDNF, (beta = 0.23, t = 8.5, p = 0.001). CuZnSOD activity, duration of illness, age, and symptom severity were not found to be important factors influencing BDNF levels in this regression model (all p > 0.05). In addition, we analyzed possible correlations between BDNF and other biomarkers in the healthy control group. There were no significant correlations between BDNF and CuZnSOD (r = 0.096, p = 0.613), MnSOD (r = −0.12, p = 0.52), GPx (r = 0.23, p = 0.22), or mtDNAcn (r = −0.12, p = 0.11) in healthy controls.

## Discussion

In the present study, we have three principal findings: a) compared to healthy controls, there was a significant decrease in plasma GPx activity and BDNF levels, a significant increase in plasma MnSOD activity, but no significant increase in TSOD in acute BD patients. b) A significant positive correlation between BDNF levels and MnSOD activity was firstly identified in BD patients. c) There was no significant correlation between mtDNA content and BDNF in BD patients.

We found abnormal GPx and MnSOD activities in manic and depressive patients, indicating oxidative stress damage in acute episodes. SOD and GPx are regarded as the first line of antioxidant defense (30). Elevated MnSOD activity and an increasing trend of CuZnSOD or TSOD activity is considered as response of the organism to high levels of superoxide, while lowered activity of GPx could induce accumulation of reactive oxygen species (ROS) that could cause protein, lipid, and DNA damages (31). Total SOD and GPx activities did not show a similar trend of changes, indicating the complexity of antioxidant defenses in manic and depressive episodes. Consistent with these findings, several previous studies have demonstrated changes of antioxidant enzymes. TSOD activity was measured rather than MnSOD in previous studies. Some reports showed higher TSOD activities during depressive and manic stages, but not in euthymia (17, 32, 33). Importantly, Machado-Vieira et al. reported increased levels of TSOD activity in unmedicated patients (17). In contrast, other studies showed decreased or normal TSOD activity (34, 35). Two meta-analyses suggested, these antioxidant enzymes formed an intricate relationship, and showed no overall significant differences compared with health controls (15, 36). Despite discrepancies among these researches, most evidence pointed to a weak antioxidant capacity and oxidative imbalance in BD.

This data showed lower BDNF levels and increased MnSOD activity in acute patients. Furthermore, BDNF levels were positively correlated with abnormal MnSOD activity after adjusting for covariates, consistent with the results of positive correlations between BDNF and TSOD in BD (23), BDNF, and CuZnSOD in schizophrenia (37). It seems contradictory that there were reduced BDNF levels, increased MnSOD activities, but a positive association between BDNF levels and CuZnSOD, MnSOD activities. Actually, all BD patients were not first-episode in this study. Recurring episodes of patients influence the outcome of BD by increasing a patient’s vulnerability to subsequent episodes (38, 39). Episodic nature of bipolar disorder may be especially detrimental to maintaining systemic homeostasis, with a repeated need for adaptation and re-setting of parameters (39, 40), which may lead to high levels of oxidative stress (41, 42), and decreased BDNF levels (42, 43). Abnormal SOD activities (34, 35, 44) and BDNF levels (43, 45, 46) were proved in euthymic patients with BD. We currently could not provide a good mechanism to explain the positive association. More longitudinal clinical studies are needed to resolve this problem.

SOD is the major enzyme to scavenge ROS and reduce oxidative stress (30). MnSOD is one such antioxidant enzyme found exclusively in the matrix of mitochondria, where it has a protective effect on mitochondrial function (47). Several studies have also confirmed the neuroprotection by MnSOD overexpression (48–51). Animal studies indicated that BDNF administration could increase the expression or production of antioxidant enzymes including MnSOD, and it may work through activating transcription factors such as NFκB, specificity protein 1, and activating protein 1 that could upregulate MnSOD expression (22, 52–55). Katusic suggested that BDNF might selectively unregulate MnSOD expression (22). Some studies also described significant relationships between oxidative stress parameters and BDNF in individuals with BD and schizophrenia (23, 37). Despite the lack of consistency, these results all suggested an interrelationship between neurotrophic and antioxidant processes in BD. More research using preclinical and clinical studies is needed to verify and clarify the relationships, especially in BD.

This data showed no correlation between BDNF and mtDNA content, an important parameter reflecting the activity of mitochondrial function and biogenesis. Mitochondrial dysfunction has been demonstrated to play important roles in BD, and may be a central feature of BD. A significant decreased mtDNAcn levels in manic patients was found in this study. The change of mtDNAcn was discussed in our previous report (28) and confirmed in later studies (56, 57). To the best of our knowledge, this is the first study to explore the relationship between BDNF and mtDNAcn in BD. Contrary to our expectations, BDNF was not associated with mtDNAcn levels. Emerging studies have shown that the effects of BDNF on mitochondria function were the same molecular pathway of its neuroprotective effect, mediated by the classic trkB-MEK and Bcl-2/Bcl-xL pathways, resulting in increased brain mitochondrial respiratory coupling at complex I (19). MnSOD is vital for mitochondrial integrity and function, and may interact with BDNF, hinting that BDNF may affect mitochondrial function. The link between BDNF and mitochondrial function may be independent of the changes of mtDNAcn, but this warrants further investigation.

In addition, we found that CuZnSOD and TSOD activities were significantly correlated with HAMD scores in depressive group. After controlling for confounding factors, the negative association between CuZnSOD, TSOD, and depressive symptom scores remained significant. Similar results were also found in previous studies (58, 59). Meta-analysis pointed out that antioxidant levels were lower in patients with major depressive disorder. Furthermore, the antioxidant levels were increased when depressive symptoms were relieved after antidepressant medication (59), suggesting that changes of antioxidant levels could be associated with the severity of depressive symptoms. Accumulating evidence revealed that using antioxidants as a therapeutic strategy for BD was beneficial in the treatment of depressed BD patients (60–62). The mechanism by which antioxidant enzymes were correlated with depression may be related to the hyperactivity of the HPA axis, which has been well noticed in depression (63–65). A recent meta-analysis of HPA axis activity conﬁrmed that BD was associated with abnormalities of stress-related pathways in the brain (66). Activation of HPA axis activity is related to imbalance of redox system and produces deleterious reactive oxygen species (67). The burden of aggravating free radicals may induce an increase in antioxidant enzymes activity, which may be involved in the pathophysiology of this disease.

There were several limitations to be mentioned in this study. First, some important factors regarding physical exercise, metabolic parameters (i.e., impaired glucose metabolism), as well as lifestyle (i.e., smoking and alcohol consumption) were not available in the present study. Second, age is an important factor that may have an impact on these biological parameters. Oxidative stress, BDNF, and mtDNAcn were reported to be associated with age in patients with BD (68–71). The biomarkers were only measured in young subjects with BD and healthy controls in this study, rather than comparisons in all age groups. Therefore, the influence of age on these results in this study can not be excluded. Third, the impact of these different kinds of medications on biochemical parameters could not be examined in this study, and no definite conclusion was made in previous studies. Yi et al. reported that SOD activities were not altered after 6 weeks of lithium treatment (72), but Selek et al. found opposite results in a similar study (34), suggesting that more research is needed in future. Similar inconsistent conclusions have also been reported in mtDNA copy number (70, 73). A meta-analysis showed that there were small increases in peripheral BDNF levels after pharmacological treatment for manic episodes, and no significant changes before and after treatment for depressive episodes (8). Based on the available evidence, the effect of drug treatment on the biomarkers can not be ignored. Fourth, this was a cross-sectional study and our sample size was small, so this study could not draw any conclusions on causality. Further prospective investigations with large samples are required to further determine the interrelationship between oxidative stress parameters, mitochondrial function, and BDNF in BD.

In summary, oxidative stress, abnormal mtDNAcn, and decreased BDNF levels may be involved in the complicated pathophysiology of BD. We observed an independent positive correlation between MnSOD activity and BDNF levels. CuZnSOD and TSOD activities were also significantly associated with HAMD scores in depressive BD patients. This study supported our hypothesis that oxidative stress, mitochondrial function, and BDNF might influence each other, indicating diverse biological systems could be involved in the pathophysiology of BD. The exact biological pathways and the role of our findings remain largely unknown in BD. Better controlled longitudinal clinical studies and fundamental research need to be conducted in future.

## Data Availability Statement

The datasets analyzed in this article are not publicly available. Requests to access the datasets should be directed to wd198529@163.com.

## Ethics Statement

The studies involving human participants were reviewed and approved by ethics committee of Suzhou Guangji Hospital. The patients/participants provided their written informed consent to participate in this study.

## Author Contributions 

DW and HL wrote the first draft of this manuscript, and HL edited the subsequent versions. HL, XD, and HR are responsible for the data collection and analysis. JZ and LY gave critical revision for the manuscript. XC and DW were responsible for the designing the study. All authors contributed to the article and approved the submitted version.

## Funding

This study was supported by the social development program of Wuxi (CSE31N1724), the Key Diagnosis and treatment Program of Suzhou (LCZX201615), National Natural Science Foundation of China (81801325).

## Conflict of Interest

The authors declare that the research was conducted in the absence of any commercial or financial relationships that could be construed as a potential conflict of interest.
