# Supplementary material for: Circulating Brain-Derived Neurotrophic Factor, Antioxidant Enzymes Activities, and Mitochondrial DNA in Bipolar Disorder: An Exploratory Report
Source: Front Psychiatry. 2020 Sep 11;11:514658. doi: 10.3389/fpsyt.2020.514658 (PMC7518036; doi:10.3389/fpsyt.2020.514658)
Supplement: Supplementary file 1 [file DataSheet_1.docx]

Supplementary Results

Table 3. Correlations of the biomarkers and clinical symptoms in BD patients

|  | BDNF | | CuZnSOD | | MnSOD | | TSOD | | MtDNAcn | | GPx | | |
| --- | --- | --- | --- | --- | --- | --- | --- | --- | --- | --- | --- | --- | --- |
| Manic Group | r | *p* value | r | *p* value | r | *p* value | r | *p* value | r | *p* value | | r | *p* value |
| YMRS scores | 0.012 | 0.93 | -0.061 | 0.67 | 0.14 | 0.34 | 0.027 | 0.85 | -0.11 | 0.42 | 0.03 | | 0.85 |
| CGI-BP-S scores | -0.044 | 0.76 | -0.18 | 0.19 | -0.015 | 0.92 | -0.18 | 0.19 | -0.1 | 0.5 | 0.027 | | 0.85 |
| Depressive group | |  |  |  |  |  |  |  |  |  |  | |  |
| HAMD scores | 0.04 | 0.97 | **-0.352** | **0.017*** | -0.08 | 0.61 | **-0.33** | **0.026*** | -0.23 | 0.13 | -0.003 | | 0.98 |
| CGI-BP-S scores | -0.044 | 0.76 | -0.19 | 0.19 | -0.015 | 0.92 | -0.23 | 0.13 | -0.098 | 0.5 | 0.027 | | 0.85 |
| MnSOD, manganese superoxide dismutase; CuZnSOD, copper zinc superoxide dismutase; TSOD, total SOD; GPx, glutathione peroxidase; BDNF, brain-derived neurotropic factor; mtDNAcn, mitochondrial DNA copy number; HAMD, Hamilton Depression Rating Scale; CGI-BD-S, Clinical Global Impression-Bipolar Disorder-Severity of Illness Scale; BD, bipolar disorder. using Partial correlation test，*P < 0.05. | | | | | | | | | | | | | |
