# Supplementary material for: Circulating Brain-Derived Neurotrophic Factor, Antioxidant Enzymes Activities, and Mitochondrial DNA in Bipolar Disorder: An Exploratory Report
Source: Front Psychiatry. 2020 Sep 11;11:514658. doi: 10.3389/fpsyt.2020.514658 (PMC7518036; doi:10.3389/fpsyt.2020.514658)
Supplement: Supplementary file 2 [file DataSheet_2.docx]

**Supplementary Methods**

**BDNF assay using an enzyme-linked immunosorbent assay (ELISA)**

The plasma concentration of BDNF was measured using a commercial ELISA kit according to the manufacturer's instructions (Mlbio, Shanghai, China) with minor modifications. Before measuring BDNF in plasma samples, we conducted a pilot study to determine if the concentrations of BDNF were within the typical range of the standard curve according to the manufacture’s instructions. Based on the values of BDNF, we used a range between 1.56 ng/ml and 50 ng/ml for the determination of BDNF in our samples. All values of BDNF in our samples were included in this range. Calculations of BDNF concentrations in the samples were determined by nonlinear regression from the standard curve included for each microplate analysis. The assay sensitivity threshold was 0.1ng/ml. The inter-assay coefficient of variation (CV) was < 7% and the intra-assay CV was < 6%, which were both below the level specified by the manufacturer. Measurements were performed in duplicated and averaged to give a mean value for each sample and expressed in ng/ml. To minimize assay variance, all the measurements were conducted on the same day.

**SOD and GPx activity assays using the spectrophotometric method**

TSOD enzyme activation was measured using a commercially available assay kit (Mlbio, Shanghai, China), with an autoanalyzer reading at 550 nm and 37°C (1). Ten µl was taken from an erythrocyte suspension and mixed with 2,500 µl of 0.01 M phosphate buffer (pH = 7.0). Later, it was diluted 251 times with water. The activity of MnSOD was measured in the same way after the addition of potassium cyanide, and the activity of cytosolic SOD (CuZnSOD) was calculated as the difference in TSOD and MnSOD activities. Inhibition was obtained between 45%–50%. The enzyme activity was expressed as units/ml.

The GPx activity was measured by the method of Paglia and Valentine (2) using commercially available assay kits (Mlbio, Shanghai, China). GPx enzyme catalyzes the oxidation of glutathione. When the oxidized glutathione is reduced, NADPH is oxidized and it becomes NADP. This change was observed at 340 nm, and the activation of GPx was measured. Plasma GPx activities were measured in triplicate. The lower limit of detection of GPx activity was 10.0 U/ml. To minimize assay variance, all measurements were conducted on the same day.

**Measurement of mtDNA**

The detailed measurement of mtDNAcn was described in our previous report (3, 4). Genomic DNA was extracted following the protocol using the QIAmp DNA Mini Kit (Qiagen, Hilden, Germany). The relative mtDNAcn was determined by the fluorescence-based quantitative polymerase chain reaction (qPCR). The primers for the mitochondrial gene [NADH dehydrogenase 1 gene (ND1)] were as follows: forward, 5'-CCCTAAAACCCGCCACATCT-3' and reverse, 5-GAGCGATGGTGAGAGCTA- AGGT-3. The primers for the reference nuclear gene (reference nuclear single copy gene, HBB) were forward, 5'-GTGCACCTGACTCCTGAGGAGA-3' and reverse, 5'-CCTTGATACCAACCTGCCCAG-3'. The double-standard curves method of relative quantification PCR was employed in this study, which is an improved, simple, accurate method. PCR cycling conditions for the ND1 gene were 95°C 10 min for one cycle, 30 cycles of 95°C for 15 s, and 60°C for 1 min. The cycling condition for the HBB gene was 95°C for 10 min, 40 cycles of 95°C for 15 s, and 56°C for 1 min. Each sample was run in triplicate using 10 ng of DNA template in a 10 µL reaction volume and randomly assigned to 96-well plates. The ND1 and HBB gene PCR reactions were performed on separate plates with the same samples located at the same positions; the amounts of mtDNAcn for each sample were obtained by the ratio of ND1 copy number to HBB copy number from standard curves. The ratio was normalized to a calibrator DNA to standardize between different independent assays. The calibrator DNA sample was diluted 1:4 to construct a five-point standard curve between 0.0195 and 20 ng/µl.

Reference

1. Floyd RA. Methods in Molecular Biology, Volume 108, Free Radical and Antioxidant Protocols. Edited by Donald Armstrong. Humana Press, Totowa, New Jersey, 1998. 455 pp. Analytical Biochemistry (1999) 270(2):342-0.

2. PAGLIA DE. Studies on the quantitative and qualitative characterization of erythrocyte glutathione peroxidase. Journal of Laboratory & Clinical Medicine (1967) 70.

3. Wang D, Li Z, Liu W, Zhou J, Ma X, Tang J, et al. Differential mitochondrial DNA copy number in three mood states of bipolar disorder. BMC Psychiatry (2018) 18(1):149.

4. Li Z, Hu M, Zong X, He Y, Wang D, Dai L, et al. Association of telomere length and mitochondrial DNA copy number with risperidone treatment response in first-episode antipsychotic-naïve schizophrenia. Scientific reports (2016) 5(1):18553.
